# 2,8-Dihydroxyadenine nephrolithiasis with chronic kidney disease in children: A case report and review of literature

**DOI:** 10.1016/j.eucr.2023.102623

**Published:** 2023-11-20

**Authors:** Rizky Ramdhani, Akhmad Mustafa, Safendra Siregar, Jupiter Sibarani, Tjahjodjati Tjahjodjati, Ahmedz Widiasta, Rini Rossanti

**Affiliations:** a Urology Department, Medical Faculty Universitas Padjadjaran, Indonesia; b Pediatric Department, Nephrology Division, Medical Faculty Universitas Padjadjaran, Indonesia

**Keywords:** 2,8- dihydroxyadenine nephrolithiasis, APRT deficiency, Nephropathy

## Abstract

The incidence of nephrolithiasis in children ranges from 5 to 10% in developing countries. Etiology of nephrolithiasis in children remains largely unknown, so metabolic evaluation is indicated in all case, because of potential morbidity and recurrence. We report a case of 2,8-Dihydroxyadenine nephrolithiasis present as bilateral staghorn stone in 11 years old boy with chronic kidney disease. 2,8-Dihydroxyadenine nephrolithiasis is the result of a metabolic abnormality due to the deficiency of the enzyme adenine phosphoribosyltransferase (APRT), it is not only promote stone formation, but also induced nephropathy. Early diagnosis ensure appropriate treatment and favorable prognosis for kidney function and stone management.

## 1 Introduction

The incidence of nephrolithiasis in children ranges from 0.1% to 5%, and it is 5–10% in developing countries. Staghorn stones are quite frequent in the adult population, but in pediatric cases, only 19% present with such stones.^1^ The etiology of nephrolithiasis in children remains largely unknown. The evaluation of children who present with nephrolithiasis should be directed towards identifying physicochemical, anatomic, metabolic, and genetic factors predisposing to nephrolithiasis. Metabolic evaluation is indicated in all cases because of the potential for morbidity and recurrence.^1^^,^^2^

2,8-Dihydroxyadenine nephrolithiasis is the result of a metabolic abnormality due to the deficiency of the enzyme adenine phosphoribosyl transferase (APRT).^3^ In the absence of APRT, 2,8-Dihydroxyadenine is excreted by the kidneys. It not only promotes stone formation but also induces nephropathy due to inflammation and tubular obstruction by 2,8-Dihydroxyadenine crystals.^2^ There have been no reported cases of 2,8-dihydroxyadenine nephrolithiasis in Indonesia. Here, we present a case of 2,8-Dihydroxyadenine nephrolithiasis in the form of bilateral staghorn stones, which were managed too late, resulting in chronic kidney disease (CKD).

## 2 Case presentation

An 11-year-old boy presented with a chief complaint of decreased consciousness accompanied with a history of general weakness, nausea, and vomiting. He had a history of atypical flank pain. He was apparently healthy before this episode and had no history of fever, passing stones, cloudy urine, or hematuria. There was no history of kidney stone disease in the family.

**Fig. 1:**
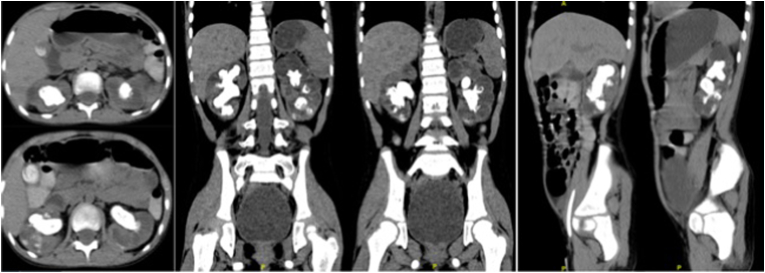
Non-contrast CT examination, showing bilateral staghorn nephrolithiasis.

Physical examination revealed facial puffiness and tachypnea. He was malnourished, and his blood pressure was 100/60 mm Hg. Laboratory investigations revealed anemia (Hb 8.8 g/dL), urinalysis showed pyuria (many leukocytes, urinary pH 7.5), chronic kidney disease (BUN 446.7 mg/dL, creatinine 7.91 mg/dL), hyperkalemia (K^+^ 5.9 mEq/L), and severe metabolic acidosis (pH 7.27, pO_2_ 66 mm Hg, pCO_2_ 21 mm Hg, HCO3- 10 mEq/L, BE -15), for which he underwent hemodialysis. Abdominal ultrasonography revealed bilateral nephrolithiasis. A non-contrast CT scan of the abdomen revealed bilateral staghorn nephrolithiasis [Fig. 1]. Kidney function revealed a left GFR of 0.396 ml/min and a right GFR of 0.252 ml/min.

**Fig. 2:**
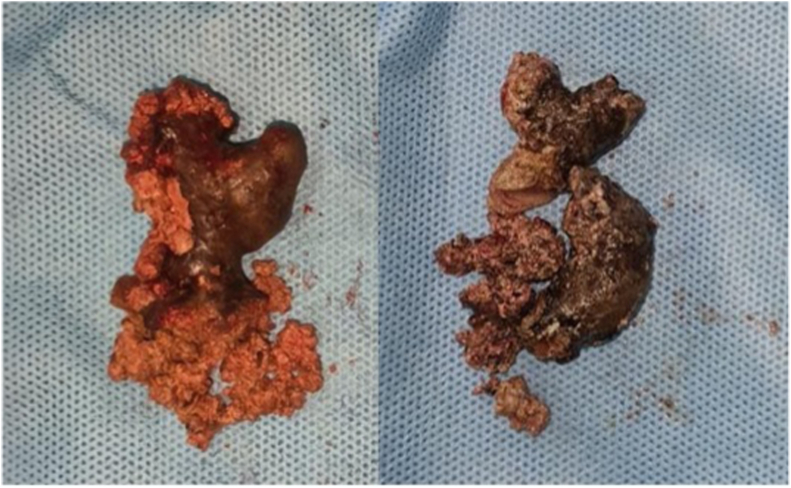
The kidney stone consists of 2,8-dihydroxyadenine, struvite, and matrix.

After improvement in the general condition in accordance with the nephrologist's recommendations, we performed bilateral nephrolithotomy. Post-operation evaluation showed no residual stones. The calculi were found to be soft, friable, and brittle with an irregular surface, and they were removed piecemeal [Fig. 2]. The stone were sent for analysis, which showed that they were composed of 2,8-Dihydroxyadenine (65%), struvite (25%), and matrix (10%). We also performed a kidney biopsy, and the result was "non-specific chronic glomerulonephritis."

After three months of follow-up, there were no complaints, but kidney function still had not improved. The patient was given allopurinol with incremental hemodialysis and prepared for kidney transplantation.

## 3 Discussion

**Fig. 3:**
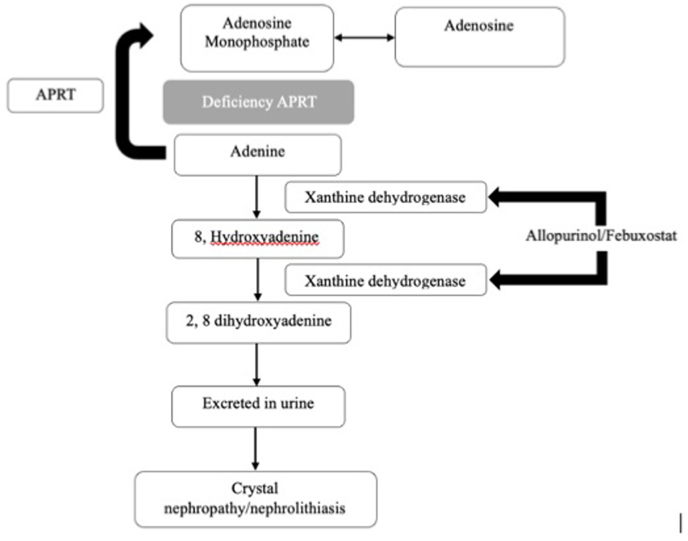
Mechanisms of adenine phosphoribosyltransferase (APRT) deficiency result in 2,8-dihydroxyadeninuria, which is insoluble in urine. Its accumulation results in crystalluria and nephrolithiasis.

This is the first case report from Indonesia describing 2.8-Dihydroxyadenine nephrolithiasis in children. 2.8-Dihydroxyadenine nephrolithiasis is caused by the deficiency of APRT, a rare autosomal recessive disorder with the gene located on chromosome 16q24.2. Deficiency of APRT results in 2,8-dihydroxyadeninuria [Fig. 3]. 2.8-dihydroxyadenine is insoluble in urine, which can accumulate, grow, and form stones or precipitate in renal parenchyma, causing crystalline nephropathy. An inflammatory reaction initiates in 2,8-dihydroxyadenine nephropathy. 2.8 dihydroxyadenine formation can be easily controlled with xanthine oxireductase (XOR) inhibitor, e.g allopurinol and febuxostat. Allopurinol administrated in a dose of 10 mg/kgBW/day in children. After treatment with allopurinol, adenine becomes the predominants purine excreted in urine instead of 2,8-dihidroxyadenine.^4^, ^5^, ^6^

In our case, 2.8-Dihydroxyadenine urolithiasis presented as bilateral staghorn stones with a composition of 65% 2.8-Dihydroxyadenine, 25% struvite, and 10% matrix. The 2.8-Dihydroxyadenine urolithiasis also acted as a nidus for struvite and matrix stones.

Initial presenting features are generally non-specific and can range from asymptomatic, hematuria, cloudy urine, flank pain, urinary tract obstruction, passing of stones, to CKD from crystal nephropathy.4 Signs and symptoms of APRT deficiency caused by stone formation in the kidney that caused obstruction, infection, or chronic kidney disease. Diagnosis of APRT deficiency can be delayed, particularly in asymptomatic nephrolithiasis patients, leading to CKD.

In our case, late admission by the parents resulted in kidney failure due to the long-standing obstruction and inflammation of the stone. Due to the lack of remaining kidney function and the complexity of the stone, we decided to perform nephrolithotomy to ensure stone clearance and avoid subsequent renal stone clearance procedures. Further follow-up is essential for the preparation of renal transplant.

Dihydroxyadenine crystals can be detected by urine microscopy, and high urine pH provides an additional clue to the diagnosis of APRT deficiency. Stone analysis is done using a stereomicroscope for morphological examination and infrared spectroscopy. If the stone is not available for analysis, the detection of 2,8-Dihydroxyadenine crystals in urine can point to the diagnosis. The gold standard methods for the diagnosis of APRT deficiency, enzyme activity measurements and genetic testing. Activity APRT measured in red cell lysates ranges from 16 to 32 nmol/hr per mg hemoglobin in healthy individuals.^4^

In our case, we did not perform DHA crystal urine examination and genetic testing because we do not have the necessary facilities for such examinations. We suggest that annual urinary screening in selected cases might be useful to prevent such late CKD conditions.

## 4 Conclusion

2,8-Dihydroxyadenine urolithiasis caused by APRT deficiency is a rare autosomal recessive disorder with significant implications for long-term kidney function and recurrent urolithiasis. Early diagnosis ensures appropriate treatment and a favorable prognosis for long-term renal function and stone management.

## Funding

This research received no specific grant from any funding agency in the public, commercial, or not-for-profit sectors.

## Declaration of Competing Interest

The authors report no declarations of interest.
